# Morphological and Molecular Basis of Cytoplasmic Dilation and Swelling in Cortical Migrating Neurons

**DOI:** 10.3390/brainsci7070087

**Published:** 2017-07-19

**Authors:** Yoshiaki V. Nishimura, Yo-ichi Nabeshima, Takeshi Kawauchi

**Affiliations:** 1 Division of Neuroscience, Faculty of Medicine, Tohoku Medical and Pharmaceutical University, 4-4-1 Komatsushima, Aobaku, Sendai, Miyagi 981-8558, Japan; yvnishimura@tohoku-mpu.ac.jp; 2 Laboratory of Molecular Life Science, Institute of Biomedical Research and Innovation, Foundation for Biomedical Research and Innovation, 2-2 Minatojima-Minamimachi Chuo-ku, Kobe 650-0047, Japan; nabemr@lmls-kobe.org (Y.-i.N.); 3 Department of Physiology, Keio University School of Medicine, 35 Shinanomachi, Shinjuku-ku, Tokyo 160-8582, Japan

**Keywords:** dilation, swelling, radial migration, tangential migration, locomotion, cerebral cortical development, Cdk5, p27, Dcx, dynein

## Abstract

During corticogenesis, neuronal migration is an essential step for formation of a functional brain, and abnormal migration is known to cause various neurological disorders. Neuronal migration is not just a simple movement of the cell body, but a consequence of various morphological changes and coordinated subcellular events. Recent advances in in vivo and ex vivo cell biological approaches, such as in utero gene transfer, slice culture and ex vivo chemical inhibitor techniques, have revealed details of the morphological and molecular aspects of neuronal migration. Migrating neurons have been found to have a unique structure, dilation or swelling, at the proximal region of the leading process; this structure is not found in other migrating cell types. The formation of this structure is followed by nuclear deformation and forward movement, and coordination of this three-step sequential morphological change (the dilation/swelling formation, nuclear elongation and nuclear movement) is essential for proper neuronal migration and the construction of a functional brain structure. In this review, we will introduce the morphological features of this unique structure in migrating neurons and summarize what is known about the molecules regulating the dilation/swelling formation and nuclear deformation and movement.

## 1. Introduction

One of the pronounced morphological features of the mammalian cerebral cortex is the six-layer structure composed of different neural cell types. Formation of this structure requires proper regulation of neuronal migration during developmental stages. Disruption of the cortical layer structure leads to several neurological disorders that are accompanied by mental retardation and/or epilepsy. These include lissencephaly, subcortical band heterotopia (also known as double cortex syndrome) and periventricular heterotopia [1,2].

**Figure 1:**
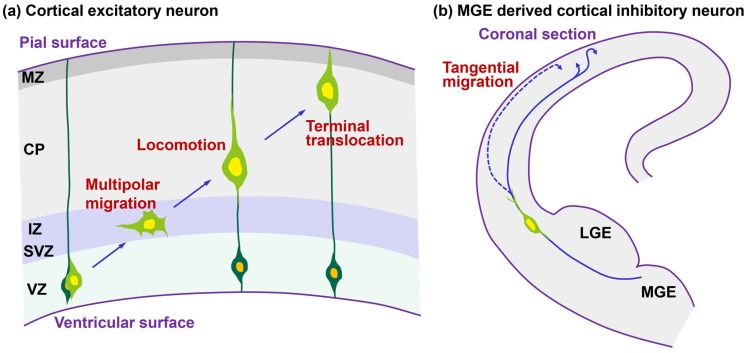
Migration mode and morphological changes of migrating neurons. (**a**) Cortical excitatory projection neurons (light green cells) are mainly generated in the ventricular zone (VZ) and transform into multipolar cells in the lower part of intermediate zone (IZ) or the subventricular zone (SVZ). Subsequently, these neurons transform into locomoting cells with a leading process in the upper part of the IZ. Locomoting cells migrate along the radial fibers of their progenitors (dark green cells) in the cortical plate (CP), and at the final term of locomotion, they contact the pial surface with the tip of the leading processes and translocate their nuclei (terminal translocation mode); (**b**) Cortical inhibitory interneurons are mainly born in the medial ganglionic eminence (MGE) and migrate tangentially through the cortex. There are three migration routes of cortical interneurons: (1) neurons migrate from the SVZ to the marginal zone (MZ) and after exhibiting non-directional movement in the MZ, they migrate into the CP, (2) neurons migrate from the SVZ to the CP, and (3) neurons directly migrate from the MGE to the MZ and subsequently migrate into the CP. Although the third route might be rare in vivo, it is frequently observed in the slice culture. LGE: lateral ganglionic eminence.

During cortical development, the progenitors of cerebral cortical excitatory neurons proliferate mainly at the ventricular zone and produce immature neurons that radially migrate toward the brain surface while undergoing various morphological changes (Figure 1a) [3,4,5,6]. Newborn neurons first enter the early phase of migration, where they exhibit multipolar morphologies and undergo several neuronal maturation events, including the acquirement of neuronal polarity and axon outgrowth in the lower part of the intermediate zone or the subventricular zone. Subsequently, they transform into bipolar-shaped neurons with a leading process, the future apical dendrite. These bipolar-shaped neurons, called “locomoting neurons”, migrate over a relatively long distance from the upper part of the intermediate zone to nearly the top of the cortical plate. At the final phase of migration, these locomoting neurons switch into the terminal translocation mode and complete the migration. Thus, neuronal migration toward the final destination is associated with neuronal maturation.

On the other hand, cortical inhibitory interneurons are generated mainly at the medial ganglionic eminence (MGE) and migrate tangentially to the cortical wall through the subventricular zone or the marginal zone (Figure 1b) [7,8,9]. The majority of interneurons enter the marginal zone directly or indirectly from the subventricular zone and exhibit multi-directional migration in the marginal zone. Subsequently, they migrate radially into the cortical plate. On the other hand, other interneurons migrate from the subventricular zone to the cortical plate without entering the marginal zone (Figure 1b). In the former case, the final radial migration of the interneurons is opposite in direction to the radial migration of the excitatory neurons, although they both migrate along the radial glial fibers.

While many neuronal maturation events occur during the early phase of neuronal migration, the locomotion mode of neuronal migration comprises most of the migration path and is therefore a main contributor to cortical layer formation [10,11]. However, as conventional methods, including gene targeting and in utero electroporation, cannot eliminate various secondary effects of defects occurring at the early phase of migration, such as the acquirement of neuronal polarity, leading process formation and multipolar migration, it has been difficult to analyze the molecular mechanisms regulating this mode. Recently, several in vitro and ex vivo techniques, allowing direct analysis of molecules involved in the locomotion mode, have been established. These include explant culture [12], slice culture-based ex vivo chemical inhibitor technique [11,13], lattice culture [14,15], co-culture of primary cortical excitatory neurons and nestin-positive cells [16], co-culture of MGE explants on dissociated cortical neurons with elongated axons [17] and cortical imprint assay [18].

Among them, the ex vivo chemical inhibitor assay is an integrated method combining an in vivo electroporation, time-lapse imaging of cortical slice culture and chemical compound screening [11]. The locomoting neurons that are visualized with in vivo electroporated fluorescence proteins are observed with a time-lapse microscopy and the cortical slices are treated with a chemical compound, such as an inhibitor for a molecule of interest, during the time-lapse observation. Using this method, the migration speed and morphological changes of the same locomoting neurons before and after the inhibitor treatment can be analyzed.

Cyclin-dependent kinase 5 (Cdk5) is a key molecule for neuronal migration [19,20] and suppression of Cdk5 by conventional methods causes defects in the early phase of neuronal migration [21,22,23]. By using the ex vivo chemical inhibitor assay, Cdk5 was shown to also regulate the locomotion mode of neuronal migration [11]. In addition, while in vivo knockdown of Fyn, a major neuronal Src kinase, disturbs the early phase of migration [11,24], treatment of cortical slice tissues with an inhibitor for Src family kinases reduces the migration speed of the locomoting neurons [11].

## 2. Dilation/Swelling Formation

### 2.1. Morphological Features of Dilation and Swelling in Migrating Neurons

Locomoting neurons show neuron-specific unique migration features [12,13,17]. (1) Locomoting neurons extend a leading process and form a cytoplasmic “dilation” (also known as a “swelling”) at the proximal region of their leading process; (2) The nuclei in the locomoting neurons elongate to enter the cytoplasmic dilation.

**Figure 2:**
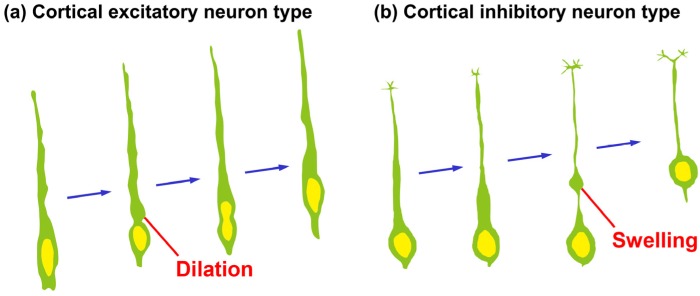
Morphological changes of leading processes of migrating neurons. (**a**) Cortical projection neurons extend a leading process and form a cytoplasmic “dilation” at the proximal region of a leading process, and the nucleus in the locomoting neurons becomes elongated to enter the cytoplasmic dilation; (**b**) Cortical interneurons extend a leading process, a cytoplasmic swelling is formed at the relatively distal region of the process and subsequently the nucleus enters the cytoplasmic swelling.

The cytoplasmic dilation or swelling was first identified in 2005 as a migrating neuron-specific subcellular domain [12,17], and is not observed in other non-neuronal migrating cells as well as static neurons [12]. The dilation was transiently formed just adjacent to the soma in the migrating neurons derived from the explants of the postnatal subventricular zone [12] and in the excitatory neurons in the developmental cerebral cortex (Figure 2a) [13,25]. In 2005, the same year when the dilation was firstly reported, Metin et al. found a similar structure in inhibitory neurons and named it a “swelling” [17]. The swelling is formed at a slightly higher position relative to the soma in inhibitory neurons of the cerebral cortex (Figure 2b). It is unclear whether the dilation in the excitatory neurons and the swelling in the inhibitory neurons have distinct characters, other than the positions at which they are formed, but at least some molecules, including p27^kip1^, differentially regulate the dilation and swelling, as described below [13,26]. Although the terminology requires further consideration in the future, in this review, we will refer to these structures in cortical excitatory and inhibitory neurons as “dilation” and “swelling”, respectively.

**Figure 3:**
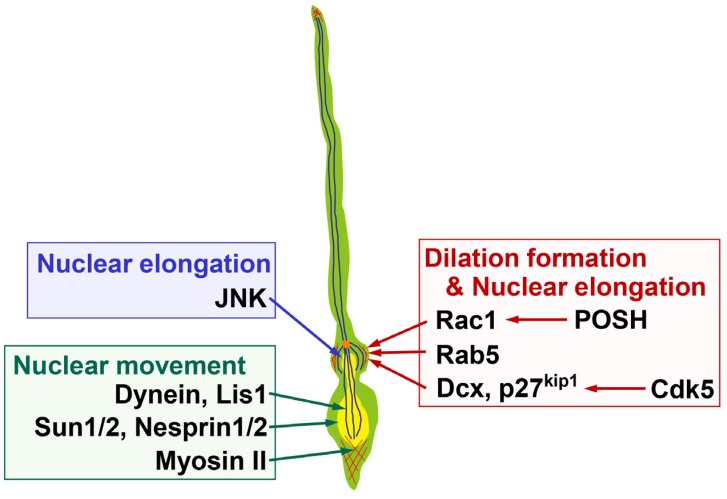
Molecules regulating the dilation formation and nuclear deformation in locomoting neurons. POSH-mediated regulation of Rac1 localization and its downstream actin reorganization, Rab5-mediated endocytic pathways and Cdk5-Dcx/p27^kip1^ pathways, regulate dilation formation. JNK, as well as POSH-Rac1, Rab5, Cdk5-Dcx/p27^kip1^ are required for nuclear elongation. Dynein/Lis1, Sun1/2, Nesprin1/2 and Myosin II control nuclear movement. Yellow and orange circles indicate the nucleus and centrosome, respectively. Blue and red lines indicate microtubules and F-actin, respectively. See the text for more details.

Electron microscopy studies show that the dilation/swelling includes the centrosome, Golgi apparatus, clathrin-coated pits and microtubules [12,17,27]. Furthermore, actin filaments are concentrated at the dilation/swelling of the migrating neurons in the developing cerebellum and cerebral cortex [13,28,29]. While treatment with cytochalasin D or latrunculin B, inhibitors for actin polymerization, disrupts the tissue structure of cultured cortical slices and makes it difficult to gauge the requirement for actin polymerization in the dilation formation, treatment with nocodazole, which inhibits microtubule polymerization, disturbs the dilation formation of the locomoting neurons [13]. In addition, treatment with dynasore, an inhibitor for dynamin-mediated endocytosis, or transfection with shRNA for Rab5, which controls endocytosis and trafficking to the early endosomes, causes defects in the dilation formation (Figure 3) [13]. These observations suggest that the dilation/swelling formation requires coordinated cellular events, including cytoskeletal organization and membrane trafficking.

### 2.2. Molecular Mechanisms Underlying Dilation and Swelling Formation

A dynein complex, a microtubule minus end-directed motor, and microtubule-associated proteins, including MAP1B and Dcx, are reported to play essential roles in neuronal migration [25,30,31,32]. Knockdown of Dcx in rat brain disturbs neuronal migration, resulting in the formation of subcortical band heterotopia, similar to the human disease with a heterozygous mutation in the *dcx* gene [32], although the knockdown of Dcx may exhibit an off-targeting effect in mice [33]. Nevertheless, Dcx is required for neuronal migration, because Dcx-knockout neurons display delayed neuronal migration [34]. It has been reported that the average migration speeds of Dcx-knockout and Dcx-knockdown locomoting neurons are 69% and 66% of control neurons, respectively, and the Dcx-knockdown locomoting neurons exhibit reduced dilation formation [13,34]. In contrast, knockdown of the dynein heavy chain or Lis1, a dynein complex-associated protein, has little effect on the dilation formation, whereas it decreases the migration rate of cortical neurons [25]. This suggests that a Dcx-mediated cellular event(s) is required for the dilation formation, whereas dynein motor activity is dispensable (Figure 3). However, Dcx controls not only microtubule organization but also membrane trafficking and actin organization [35,36,37,38], and it is unclear which downstream event(s) mediates Dcx-dependent regulation of the dilation formation.

Cdk5 is known to directly phosphorylate Dcx [39]. Cdk5-mediated phosphorylation of Dcx reduces its microtubule-binding affinity [39]. Pharmacological or knockdown-mediated inhibition of Cdk5 suppresses the dilation formation (Figure 3) [13]. Consistent with the fact that suppression of Cdk5 increases the Dcx activity to control microtubule organization, overexpression of Dcx weakly suppresses the dilation formation of the locomoting neurons. In addition, knockdown of Dcx partially, but not fully, rescues the defects in the dilation formation in Cdk5-knockdown locomoting neurons [13]. Thus, both insufficient and excess Dcx lead to a similar phenotype, suggesting that proper regulation of Dcx activity by Cdk5 plays an important role in the dilation formation.

The fact that knockdown of Dcx cannot fully rescue the phenotypes of Cdk5-knockdown implies that (an)other downstream molecule(s) also play(s) a role in the dilation formation. To date, many downstream substrates have been identified [40]. Among them, p27^kip1^ is reported to be phosphorylated by Cdk5 at Ser10. This phosphorylation protects it from proteasome-dependent protein degradation, resulting in stabilization of the p27^kip1^ protein [22]. Knockdown of p27^kip1^ is shown to suppress dilation formation and the locomotion mode of the migration of cortical excitatory neurons (Figure 3) [13]. In contrast, suppression of p27^kip1^ in cortical inhibitory interneurons promotes the swelling formation [26], implicating the molecular differences in the dilation and swelling formation.

While p27^kip1^ is a CDK inhibitory protein and controls G1 length and cell cycle exit in the nucleus [41,42,43], recent studies have indicated that p27^kip1^ also functions in cytoplasm to regulate both actin and microtubule organization [22,26]. Although it is unclear which downstream event(s) of p27^kip1^ is important for the dilation formation, Cdk5 is thought to regulate both actin cytoskeleton and microtubules. This is because suppression of Cdk5 results in a decrease of actin accumulation at the dilation as well as perturbation of the Golgi positioning, which largely depends on microtubules and dynein motor activity, in the locomoting neurons [13]. Furthermore, knockdown or pharmacological inhibition of Cdk5 disturbs the microtubule organization in primary cortical neurons [13].

Actin organization is mediated by Rho family small GTPases, such as RhoA, Rac1 and Cdc42 [44,45]. While RhoA negatively controls neuronal migration [46] and is suppressed by Cdk5 and its downstream molecules, p27^kip1^ and Mst3 [22,47], Rac1 promotes neuronal migration; suppression of Rac1 by the expression of a dominant negative form or a knockdown vector or conditional gene targeting results in delayed or stalled neuronal migration [21,28,48,49]. While mDia proteins, downstream effectors of RhoA, are reported to regulate centrosomal positioning and nuclear translocation in tangentially migrating cortical inhibitory neurons, mDia deficiency does not impair the swelling formation [50]. In contrast, a scaffold protein, POSH, enhances the plasma membrane localization of an activated form of Rac1 and suppression of POSH or Rac1 reduces dilation formation (Figure 3) [28]. Furthermore, knockdown of POSH disturbs the actin accumulation at the dilation, suggesting that POSH and Rac1 promote the dilation formation through the regulation of actin organization [28]. It is known that Rac1 activates JNK, which regulates the leading process morphology and neuronal migration [21]. However, suppression of JNK does not affect the cross-sectional area of the dilation in the locomoting excitatory neurons, although JNK-suppressed neurons form an irregular shaped dilation, suggesting that regulation of dilation formation by Rac1 occurs mainly in a JNK-independent manner [13].

What is an environmental cue for the dilation/swelling formation? Although the migration of both excitatory and inhibitory neurons requires N-cadherin in vivo [16,51,52], cultured inhibitory interneurons migrating on a biomimetic N-cadherin substrate (but not cadherin-6, cadherin-11, E-cadherin and laminin substrates) exhibit fast migration in a non-saltatory fashion and have difficulty in forming the swelling, suggesting the importance of a balanced extracellular environment [52,53]. While radially migrating excitatory neurons in vivo form the dilation [13], an in vivo imaging study indicates that the swelling is rarely observed in cortical interneurons in the MZ, where the interneurons frequently change their migration direction unlike tangentially migrating interneurons from the MGE [54]. These observations suggest that an extracellular environment controls the dilation/swelling formation, which may be associated with directional and/or saltatory movement of the locomoting neurons.

## 3. Nuclear Deformation and Movement

### 3.1. Nuclear Deformation during the Locomotion

After the formation of the cytoplasmic dilation or swelling, the locomoting neurons deform their nuclei. Nuclear sphericity is transiently decreased and, accordingly, the ratio of the length to width of the nuclei is increased. The elongated nucleus moves forward and enters the dilation/swelling, and as a consequence, the dilation/swelling is replaced by the nucleus. Subsequently, the locomoting neurons form another dilation/swelling. This cycle of dilation/swelling formation, nuclear elongation and nuclear forward movement is repeated and results in the saltatory movement of the locomoting neurons [12,13,17].

Such a nuclear deformation is also observed in non-neuronal cells migrating through confined spaces. Cells migrating through dense extracellular matrix (ECM) transiently exhibit hourglass-like or cigar-shaped elongated morphologies [55,56]. Furthermore, real-time in vivo imaging revealed that cancer cells injected in the heart of nude mice display elongated nuclei during migration in the capillaries of the living mice [57]. The dilation/swelling formation, however, has never been observed in migrating non-neuronal cells [12]. Thus, the cytoplasmic dilation or swelling is a migrating neuron-specific unique structure, but the nuclear deformation is a broadly observed event in many cell types.

### 3.2. Molecules Regulating Nuclear Elongation

From the molecular aspect, the nuclear deformation is related to the cytoplasmic dilation formation. Suppression of Cdk5, Dcx, p27^kip1^ or Rab5 perturbs the nuclear elongation as well as dilation formation during the locomotion mode [13]. Involvement of actin rearrangement in the nuclear elongation of the locomoting neurons is unclear, whereas the nuclear deformation of non-neuronal cells requires several actin-binding proteins, including Arp2/3 complex and Fascin [58,59]. In contrast, treatment with nocodazole suppresses the nuclear deformation in neurons, suggesting that microtubule organization plays a role in the nuclear elongation of the locomoting neurons [13]. Previous observations reveal that cage-like microtubules surround the elongated nuclei in neurons [12,60,61], and Cdk5-mediated phosphorylation of focal adhesion kinase (FAK) at Ser732 is reported to control the perinuclear cage-like microtubule organization and nuclear elongation [62].

While JNK does not affect the cross-sectional area of the cytoplasmic dilation, knockdown of JNK suppresses the nuclear elongation (Figure 3), suggesting that Cdk5 and JNK, both of which are known to control microtubule dynamics [21,30], have different roles in the locomotion mode [13]. Although it is unclear whether JNK activation can occur downstream of Cdk5, suppression of Rac1 or Rab5, which disturbs the dilation formation, decreases the activity of JNK in cortical neurons [16,21], implying that JNK might be a factor connecting dilation formation and nuclear elongation.

In line with this, an interesting question is whether the dilation/swelling formation and nuclear deformation are independently regulated or whether the dilation/swelling formation induces the nuclear deformation. As described above, suppression of many of the molecules involved in the dilation formation leads to defects in the nuclear elongation. However, time-lapse imaging revealed that treatment with an inhibitor for CDKs, including Cdk5, quickly suppresses the dilation formation but gradually increases nuclear sphericity as the nuclear movement is inhibited [13]. Thus, a primary target of Cdk5 may be dilation formation, and defects in nuclear deformation in the Cdk5-suppressing locomoting neurons may be secondary to abnormalities in the dilation formation or nuclear movement. This may explain why most of the molecules analyzed in the previous studies are involved in both the dilation formation and nuclear deformation.

On the contrary, the fact that migrating non-neuronal cells exhibit nuclear deformation without forming the dilation or swelling may support the former hypothesis; dilation/swelling formation and nuclear elongation are independent. In fact, Cdk5 may regulate dilation formation and nuclear deformation through different downstream pathways; the Dcx- and p27^kip1^-mediated [13] and the FAK-mediated [62], respectively. It may be important to identify events upstream of JNK activation, because it may provide a clue clarifying whether dilation formation induces the JNK-mediated nuclear deformation or whether other molecular pathways activate it in a dilation formation-independent manner.

### 3.3. The Forward Movement of the Nucleus

Considering that the elongated (migrating) nuclei are surrounded by the cage-like microtubules, perinuclear microtubules are thought to regulate the nuclear forward movement. In fact, suppression of dynein or Lis1 results in abnormally increased distance between the centrosome and nuclei, but has no effect on the dilation formation, suggesting that retrograde transport on microtubules plays an important role in nuclear movement [25,39]. PDK1 and its downstream molecule, Akt, have been shown to promote the somal migration speed of the locomoting neurons through the regulation of coordinated movement of the nucleus and centrosome [63]. Although PDK1 may not directly regulate the nuclear movement, PDK1 is required for the binding of Lis1 and p150^Glued^, a subunit of the dynein–dynactin complex, to microtubules and promotes the formation of perinuclear cage-like (mesh-like) microtubules and the centrosomal movement [63].

Given that dynein-mediated microtubule retrograde motor activity is involved in the nuclear movement, it requires a factor that would link the nucleus with the perinuclear microtubules. SUN-domain proteins, Sun1 and Sun2, and KASH-domain proteins, Nesprin-1 and Nesprin-2, are components of the linker of the nucleoskeleton and cytoskeleton (LINC). Sun1/2 are transmembrane proteins that are localized at the inner nuclear membrane of the nuclear envelope and directly bind to A- and B-type nuclear lamins, whereas Nesprin1/2 are transmembrane proteins spanning the outer nuclear membrane and interact with the dynein–dynactin complex [64,65,66]. Sun1/2- or Nesprin1/2-deficient mice show defects in both nuclear movement and centrosome-nucleus coupling in cortical neurons [64]. Lamin B2-deficient mice also exhibit neuronal migration defects in the developing cerebral cortex [67]. Thus, the SUN and KASH proteins function to link the nucleus to the microtubules and are required for proper nuclear movement in migrating neurons (Figure 3).

In addition to microtubules, actomyosin-mediated contractility is known to play an important role in the nuclear forward movement (Figure 3). A study using a traction force microscopy shows that migrating cerebellar granule neurons have three contraction centers at the distal and proximal regions of the leading process and the posterior end of the neurons [68]. Actomyosin-mediated contractility at the posterior end of the migrating neurons, where activated myosin II is observed, pushes up the nuclei in the migrating neurons [12,69]. Myosin II is also observed at the proximal region of the leading process and controls the coordinated movement of the centrosome and soma in migrating cerebellar granule neurons [29]. In addition, local perfusion with latrunculin A, an F-actin depolymerizing agent, at the leading process, but not the soma, suppresses the nuclear movement in cultured cerebellar granule neurons [70]. On the other hand, in the locomoting neurons in the developing cerebral cortex, treatment with blebbistatin, an inhibitor for myosin activity, or knockdown of myosin IIB blocks the nuclear forward movement, but has no effect on the centrosomal movement [25]. At any rate, actin polymerization and actomyosin-mediated contractility are important for the nuclear movement of the migrating neurons, whereas the involvement of actomyosin contractility in the centrosomal movement may depend on neuronal subtypes.

The accumulation of actomyosin is controlled by an Elongator complex that is known to regulate the acetylation of tubulin in neurons and proper tRNA modifications in neural progenitors [71,72]. Conditional deletion of Elp3, an enzymatic core subunit of the Elongator complex, in cortical inhibitory interneurons increases the depolymerization of actin filaments and perturbs the accumulation of actomyosin at the nuclear rear or trailing process, resulting in reduction of the nuclear migration velocity [73]. Interestingly, deficiency of Elp3 suppresses the swelling formation in cortical inhibitory interneurons, suggesting its multiple roles in coordinated neuronal migration.

## 4. Conclusions

Morphological changes of migrating neurons are essential for proper brain formation. Recent advances in in vivo cell biological approaches have helped to uncover the unique features of neuronal migration; the dilation/swelling formation and nuclear deformation. However, the differences in the mechanisms regulating the dilation and swelling formation and the correlation between the dilation/swelling formation and nuclear deformation remain to be further clarified. Future studies to identify mechanisms regulating the dilation/swelling may shed more insight into the neuronal migration during cortical development.
